# Cannabinoid Neuromodulation in the Adult Early Visual Cortex

**DOI:** 10.1371/journal.pone.0087362

**Published:** 2014-02-19

**Authors:** Ifije E. Ohiorhenuan, Ferenc Mechler, Keith P. Purpura, Anita M. Schmid, Qin Hu, Jonathan D. Victor

**Affiliations:** Division of Systems Neurology and Neuroscience, Brain and Mind Research Institute, Weill Cornell Medical College, New York, New York, United States of America; University of Southern California, United States of America

## Abstract

Sensory processing is an active process involving the interaction of ongoing cortical activity with incoming stimulus information. However, the modulators and circuits involved in this interaction are incompletely understood. One potential candidate is the cannabinoid-signaling system, which is known to modulate the dynamics of cortical networks. Here, we show that in the primate primary and secondary visual cortices, the cannabinoid CP55940 modulates not only population dynamics but also influences the dynamics of the stimulus-response relationship of individual neurons. At the population level, CP55940 decreases EEG power, LFP power, and LFP coherence. At the single-neuron level, intrinsic spike train dynamics appear relatively unchanged, but visual receptive fields are altered: CP55940 induced an overall delay and broadening of the temporal component of V1 and V2 spatiotemporal receptive fields. Our findings provide neurophysiologic evidence for a link between cannabinoid-signaling, network dynamics and the function of a canonical cortical circuit.

## Introduction

The processing of sensory information by the neocortex is thought to arise from an interaction between the highly recurrent networks in local cortical circuits and the afferent streams of activity arising from sensory pathways. Recurrent networks are often thought to be the source of background activity in the cortex. To the extent that background synaptic barrages can be regarded as noise, this activity acts as a limit on signal transmission [1]. However, there is considerable evidence to suggest that background activity may be an essential component of cortical processing, since it appears to play a crucial role in modulating neuronal gain [2], [3] and mediating the influence of higher-order processes such as attention [4], [5], [6]. As a functional consequence of this interaction between background activity and afferent drive in the sensory cortices, the representation of stimulus information in the olfactory [7], somatosensory [8] [9], and visual cortices [10] [11] is rapidly modulated by ongoing cortical activity. Despite the important role that recurrent cortical network dynamics play in stimulus encoding, the neuromodulators involved in these interactions are incompletely understood [12].

One neuromodulator that is likely to play an important role is the endocannabinoid system. The cannabinoid receptor CB1, is widely expressed in the cortex, and in the hippocampus, CB1 modulates gamma frequency oscillations in the local field potential (LFP) [13]. The role of cannabinoid signaling in network oscillations suggests that it might be involved in the interactions of incoming sensory activity and intrinsic dynamics. But despite the widespread expression of CB1 in the brain, very little is known about its role in sensory processing. The cannabinoid system is known to mediate synaptic plasticity in sensory cortices during development [14], [15], [16], [17] but, the persistent expression of CB1 in the primary visual cortex (V1) of the adult primate [18], suggests that cannabinoid mediation of sensory information occurs in the adult as well, and at the earliest stages of cortical processing. Motivated by these considerations, we asked whether a CB1 agonist could alter the dynamics of networks in the primary visual cortex and sought to determine what effect this has on stimulus encoding.

## Results

We performed extracellular recordings of single neurons using an array of tetrodes in V1 and V2 of anesthetized macaque monkeys. We used a pseudo-random checkerboard stimulus to drive these cells, while recording the electroencephalogram (EEG), local field potentials (LFP) and single-unit activity. After these baseline recordings, we systemically administered the cannabinoid receptor agonist CP55940 [13] to investigate the role of cannabinoids on network dynamics and receptive fields.

**Figure 1:**
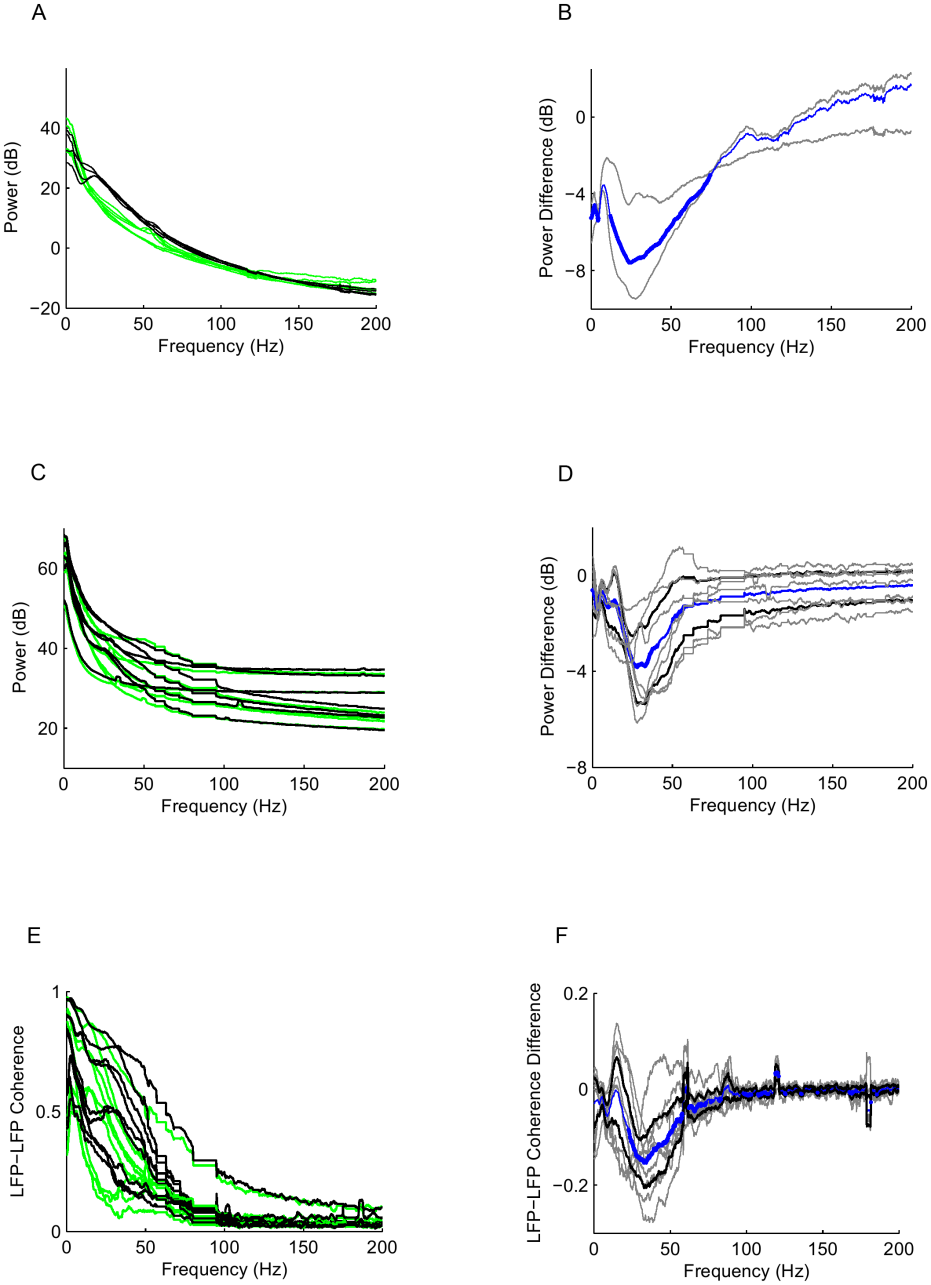
The cannabinoid CP55940 alters the dynamics of neuronal populations across a range of cortical scales. (A) Frontal EEG power spectra before (2 trials – black) and after (3 trials – green) CP55940 administration, in two animals, L65 and L68. (B) Difference (CP55940-control) in EEG power in two animals (gray), mean shown in blue; heavier segments indicate p<0.05 by two group test. (C) LFP power at 7 tetrodes in 2 animals, L69 (V1 and V2 sites) and L72 (V2 sites), before (black) and after (green) CP55940. (D) Difference (CP55940-control) in LFP power spectra (gray), mean shown in blue; heavier segments indicate p<0.05 by two group test. Black lines indicate +/−2 SEM. (E) LFP-LFP coherence from 9 tetrode-pairs (3 pairs from the three tetrodes in L69, 6 pairs from the four tetrodes in L72), as in C. (F) Difference in LFP-LFP coherence, as in D.

We recorded EEGs and LFPs in two animals each before and after cannabinoid administration. We found that CP55940 alters network dynamics across a range of cortical scales (Figure 1). First, relative to the control condition, we observed a broadband decrease in EEG power (120 Hz and below) following administration of the cannabinoid (Figure 1A and Supplementary Fig. 1A in File S1). The difference in EEG power was significant between 15–80 Hz (p<0.05, two group test) (Figure 1B). At the level of the local field potential (LFP), recordings from 7 tetrodes in two animals showed decreased activity in the low gamma band frequencies (20–50 Hz) in the presence of CP55940 (Figure 1C,D). In one animal, CP55940 decreased LFP power up to 40 Hz (Supplementary Fig. 1B in File S1– solid lines), while in the other, the LFP power was decreased over the 20–120 Hz range (Supplementary Figure 1B in File S1– dashed lines).

Since the EEG reflects global brain activity, and the LFP is driven by local synpatic input [19], these findings suggest that cannabinoids induce a widespread desynchronization of cortical networks. To examine this directly, we calculated the LFP-LFP coherence between individual tetrodes in the array. Coherence is a frequency domain measurement of the correlation between two signals, normalized to account for any changes in the spectra of the individual signals. We found that CP55940 decreases the LFP-LFP coherence between 25–60 Hz (Figure 1E,F). In one animal, cannabinoid administration decreases LFP-LFP coherence below 40 Hz (Supplementary Fig. 1C in File S1– solid lines), while in the other, the decrease in coherence occurs between 30–50 Hz (Supplementary Fig. 1C in File S1– dashed lines). Although there is some difference in the affected frequency ranges between the two animals, in both cases, the decrease in coherence occurs in a low-frequency subset of the frequency range at which CP55940 decreased LFP power (compare Supplementary Fig. 1B and 1C in File S1). Since the LFP-LFP coherence is thought to reflect shared synaptic input [19], our findings suggest that cannabinoids modulate the level of common synaptic input from distant local networks.

**Figure 2:**
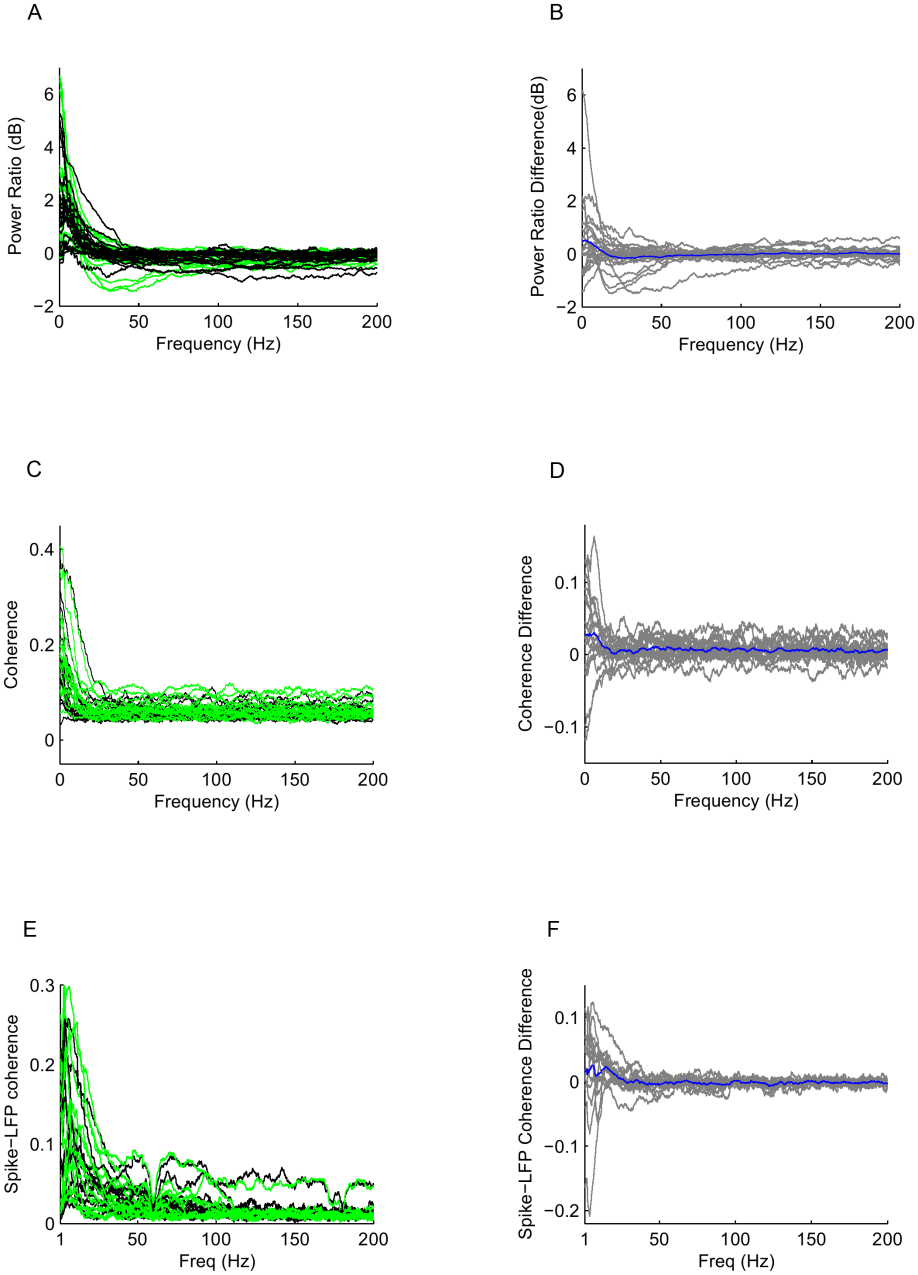
CP55940 does not alter spike train dynamics of neurons or groups of neurons. (A) Normalized spike spectrum (power ratio) of individual neurons before (black) and after (green) cannabinoid administration, across 24 cells in 5 animals. (B) Difference in power ratio (CP55940-control) for all cells (gray), mean shown in blue. (C) Coherence between 18 pairs of neurons before (black) and after (green) cannabinoid administration in 5 animals. (D) Difference in coherence, as in B. (E) Spike-LFP coherence for 12 spike-LFP pairs in 2 animals before (black) and after (green) cannabinoid administration. (F) Difference in spike-LFP coherence, as in D. None of the differences in B, D, or F were significant at p<0.05.

As a first step in examining the effect of CP55940 on neurons, we calculated the power spectrum of the spiking activity of individual neurons. We found that across 24 cells in 5 animals, there was no statistically significant difference in the spike train power spectrum following cannabinoid administration (two-tailed paired t test, p>0.05 across all frequencies, Figure 2A,B). Similarly, we did not find a significant difference in the coherence between 18 pairs of neurons (p>0.05 across all frequencies, Figure 2C,D and Supplementary Fig. 2B in File S1). To further examine the coupling between the spiking activity of neurons and their synaptic input, we measured the spike-LFP coherence, and here too, we found no significant difference before or after cannabinoid administration (p>0.05 across all frequencies, Figure 2C). Thus, while cannabinoid receptor activation decreases the synchronization of populations of neurons as measured by the EEG and LFP, these changes have little apparent effect on the intrinsic spike dynamics of individual neurons, or groups of neurons in the early visual cortex.

**Figure 3:**
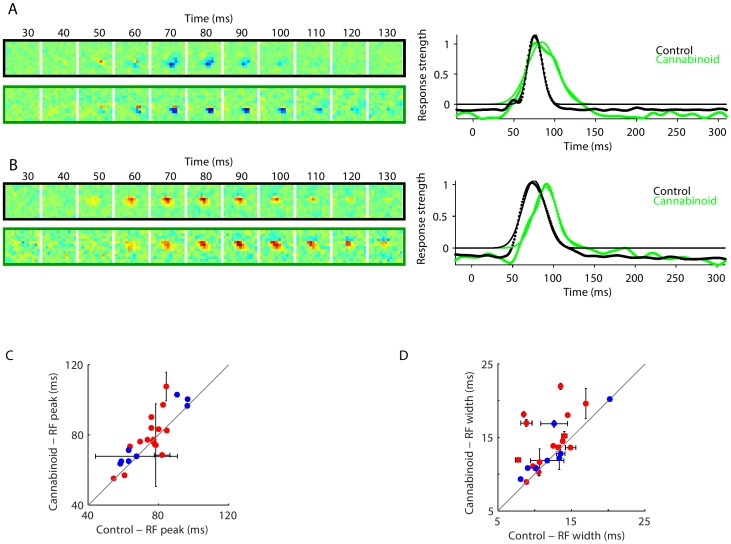
CP55940 alters V1 receptive fields. Left: examples of spatiotemporal response functions for two neurons, unit L65t1.a (A), and unit L64t2.b (B), in the control condition (top row) and following cannabinoid administration (bottom row). Right: response profile (normalized mean-square values of the spatiotemporal response function at each time point), in each condition (dots), along with Gaussian fits (thin lines). (C) Response latency and (D) response duration in control and cannabinoid conditions, determined from the mean and standard deviation of the Gaussian fits to the response profile in V1 (red) and V2 (blue) neurons. For further examples, see Supplementary Figures 2 and 3 in File S1.

To determine if cannabinoid administration was associated with a change in the stimulus-response relationship, we mapped receptive fields before and after CP55940 dosing. Figure 3A,B (left) shows the results for two example neurons. In general, CP55940 induced complex changes to neuronal receptive fields (see Supplementary Fig. 2 in File S1 for further examples) but the most robust and systematic effects were alterations in the response time course. To quantify this, we calculated the response strength (see Methods) for each neuron, as a function of time (Fig. 3A,B right). Across the population of 24 neurons with receptive fields that could be mapped by our methods, CP55940 increased the latency of the peak response (Fig. 3C, mean change of 7.7+/−3.7 ms, p = 0.049 by two-tailed paired t-test). Along with this increase in latency, the cannabinoid agonist broadened the duration of the neuronal response to visual input, as quantified by the standard deviation of the best fitting gaussian to the temporal response profile (Fig. 3D, mean of 11.9+/−2.4 ms in the control condition versus 14.1+/−3.4 ms in the CP55940 condition, p = 0.005 by two-tailed paired t-test). Additionally, in some neurons, there were changes in the spatial aspects of the receptive field structure (see Supplementary Fig. 2 in File S1, second neuron), but these changes were not typical of the population. Of note, some neurons showed no change in receptive field structure (see Supplementary Fig. 3 in File S1 for examples). Comparing cells with receptive field changes after CP55940 to those without, we found no difference in the average spike width (p = 0.5 by two tailed t-test), and the presence or absence of a change did not correlate with their classification as a fast or slow spiking cell (p = 0.4, two-tailed Fisher exact test); however, the implications of the absence of a correlation are limited due to the modest sample size.

Finally, we found that cannabinoid administration led to a decrease in firing rate (Supplementary Fig. 4 in File S1 p = 0.039, two-tailed paired t-test). This change was largely due to cells whose receptive fields were altered by CP55940 (p = 0.02); cells whose receptive fields were not altered did not show a firing rate change after the cannabinoid (p = 0.2).

In sum, we show that CP55940 modifies the encoding of visual stimuli, principally by delaying and broadening the temporal response functions of V1 and V2 neurons.

## Discussion

We examined the effect of systemic administration of a cannabinoid, CP55940, on the dynamics of neuronal populations in the early visual cortex and on receptive field structure. Although CP55490 is considered reasonably specific (Robbe et al., 2006), animal welfare concerns precluded a direct demonstration that the effects we attribute to CP44940’s action on the CB1 receptor could be reversed via CB1 antagonists. CB1 receptor antagonists can induce hyperalgesia [20], potentially interfering with our ability to provide appropriate anesthesia. Since the animals were under neuromuscular blockade (to enable detailed receptive field mapping), we would not have been able to guard against this possibility.

We found that cannabinoid administration has two significant effects. First, it leads to a broadband decrease in power in the EEG and LFP signals as well as in LFP-LFP coherence. Second, it causes a lag in the time course of individual neuronal responses, delaying them by ∼10 ms and also broadening them in time. While cannabinoids have been shown to play an important role in the development of the sensory cortices [17], [15], [16], [14], the neurophysiologic role for cannabinoids in visual processing is less clear. Although not extensively studied, a few psychophysical studies support an interaction between cannabinoids and the visual system [21]. Our findings show that at the neurophysiological level, cannabinoid signaling plays an important role in visual processing modulating activity over a range of cortical scales (globally and locally) as well as altering cortical processing at the single-cell level. We note, however, that while endocannabinoid receptors are not present in the primate thalamus [18], they are present in the primate retina [22], so we cannot rule out the possibility that endocannabinoid effects on cortical input contribute to our findings. Specifically, changes in subcortical latencies and dynamics could produce corresponding changes in cortical single-unit responses. At present, there is insufficient data to evaluate this possibility, as there appear to have been no studies of the effects of endocannabinoids on the spatiotemporal receptive field structure of precortical visual neurons.

At the level of the local field potential, the data show a reduction in gamma-band power and a reduction in coherence across a broad frequency range. Since LFP-LFP-coherence is thought to originate from shared synaptic input [19] over hundreds of microns [23], this suggests that one action of CP55940 is to desynchronize V1 by decreasing the common synaptic inputs at this cortical scale. The parallel effect on the EEG suggests that this desynchronization extends to the scale of several millimeters or more. As CB1 agonists are believed to limit the release of GABA by inhibitory interneurons in the cortex [18], the changes in the EEG, LFP and the response dynamics to afferent drive we observe here may result from an alteration in the balance of excitation and inhibition in local cortical circuits. This change in balance may not produce changes in network synchronization [24] other than to decrease shared synaptic drive within the local circuit. In this regard, we note that we found a modest decrease in firing rate in cells whose receptive fields were altered by CP55940 (Supplementary Figure 4 in File S1).

In the hippocampus, CB1 receptor activation decreases gamma oscillations [25], [26] [13]. Here, we show that cannabinoid modulation of the EEG and LFP extends to the neocortex and results in a broadband decrease in power. Intriguingly, the psychedelic drug psilocin also induces a broadband decrease in power over a similar range [27], suggesting a common mechanism of action for psychoactive drugs that alter consciousness. Our findings here also indicate that cannabinoid-signaling is involved in the interaction between stimulus input and cortical dynamics. Specifically, CP55940 leads to a delay in the response of V1 and V2 neurons to visual input as well as a broadening of their temporal response. Taken together, our findings suggest that cannabinoid-signaling is part of a mechanism to flexibly modulate the encoding of stimulus information by on-going cortical dynamics – a key feature of cortical processing [28], [12].

## Methods

### Physiological Preparation

All procedures were in accordance with the National Institutes of Health guidelines for the use and care of experimental animals and were approved by the Weill Cornell Medical College Institutional Animal Care and Use Committee. We recorded from area V1 and V2 of 5 anesthetized, paralyzed macaque monkeys (*Macaca mulatta*) using one or two three-tetrode recording arrays; inter-tetrode separation was 300 to 900 microns. Spiking activity was sampled at 22 kHz from the raw signals by bandpass filtering from 300–6000 Hz. In four animals, EEG (two animals) and LFP activity (two animals) were recorded continuously at 473 Hz. Full details of the experimental preparation have been previously provided [29]. Visual stimuli consisted of pseudorandom checkerboards which were presented at a frame rate of 100 Hz in blocks of 22 minutes, consisting of 16, 83-second long, trials. Each checkerboard consisted of a 13×13 array of checks, each subtending 0.2×0.2 degrees.

### Drugs and Injections

CP55940 (Sigma) was dissolved in ethanol to achieve a stock solution of 3 mg/ml. For each animal, a 0.3 mg/kg dose of CP55940 injection solution was prepared using a mixture of 10% stock solution, 10% Cremaphor and 80% lactated Ringer’s solution. For vehicle-only injections, an equal volume of 10% ethanol, 10% Cremaphor and 80% lactated Ringer’s was prepared. Injections were delivered intravenously through either the cubital or femoral veins.

Baseline neuronal responses were initially recorded in response to the pseudorandom checkerboard stimulus. Following this, the vehicle-only injection was administered and after 15 minutes, responses to visual stimuli were again recorded. Afterwards, a 0.3 mg/kg dose of CP55940 was delivered and 15 minutes later, three recording sessions (spontaneous activity and receptive field mapping) were completed. Each recording session lasted 22 minutes. The baseline and vehicle-only recordings were pooled to form the “control condition.” Similarly, the three post-CP55940 infusion recordings were pooled to form the cannabinoid condition. Pooling over datasets helped reduce the effect of variability in neuronal responses.

### Electrophysiologic Recording and Data Analysis

EEG recordings were made in two macaques from head screws implanted in the frontal skull at the level of the bregma, approximately 8 mm apart. Spectral analyses (power spectra and coherences) were carried out via the multitaper method, implemented by the Chronux toolbox (http://chronux.org) for Matlab (Mathworks). The EEG power spectra (Figure 1A) were estimated using 5 Slepian tapers and 4-second segments. The difference spectra (Figure 1B) were obtained by subtracting the log power (i.e., power in dB) obtained in the control condition of Figure 1A from that obtained in the cannabinoid condition. LFP recordings were obtained from each tetrode in the array (the most distal contact in each tetrode, referenced to ground), and the power spectra (Figure 1C) and difference spectra (Figure 1D) were computed in the same way as for the EEG. We recorded LFP data in 2 animals from each tetrode at which neural activity was present (3 tetrodes in L69, 4 tetrodes in L72). The average LFP power spectra shown in Supplementary Figure 1B in File S1 are the mean log power spectra across tetrodes, averaged within each condition and in each animal. Coherences between LFPs (Figure 1E) were calculated for each pair of simultaneously recorded signals (i.e., each pair of tetrodes, Figure 1C). The difference in LFP coherences (Figure 1F) were calculated by subtracting the coherence in the control condition from the coherence in the cannabinoid condition. The average LFP-LFP coherences shown in Supplementary Figure 1C in File S1 are the mean LFP-LFP coherences, averaged within each condition and in each animal. To determine the significance of differences of EEG or LFP spectra recorded under cannabinoid and control conditions (Fig. 1B, D; Supplemental Figure 1A, B in File S1), we used the two group test (a jackknife U-statistic as implemented by the Chronux toolbox) on the average EEG or LFP power spectrum in each recording session. To determine the significance of differences between LFP coherences recorded under cannabinoid and control conditions (Fig. 1F, Supplemental Figure 1C in File S1), we used a two-tailed paired t-test, pairing the values obtained at each site.

Single cell spiking activity was sorted from the raw extracellular recordings (300 to 9000 Hz bandwidth) using the KlustaKwik algorithm [30] available in SpikeSort3D (Neuralynx). Spike trains were binned into 10 ms intervals. Spike spectra between 0.1 and 500 Hz were estimated by the multi-taper method using 5 Slepian tapers and 4-sec segments, as for the EEG and LFP. As is standard, this yields a normalization in which the high-frequency asymptote of the power spectrum for each cell and each condition is equal to the mean firing rate. To facilitate a comparison across cells with different firing rates, we divided the power spectrum by the high-frequency asymptote (Figure 2A). For each cell, the power spectrum difference was calculated by subtracting the log power spectrum in the control condition from that of the cannabinoid condition (Figure 2B). We calculated the coherence between neuron pairs (Figure 2C) for all simultaneously recorded pairs of neurons (18) using Slepian tapers identical to those used for the spike spectrum. The difference in coherence (Figure 2D) was calculated by subtracting the coherence in the control condition from the coherence in the cannabinoid condition. The spike-LFP coherence (Figure 2E) and the cannabinoid vs. control difference (Figure 2F) was similarly estimated for each neuron recorded at a tetrode and the LFP recorded from that tetrode. To determine the significance of differences between spike spectra (Figure 2B), spike-spike coherences (Figure 2D) or spike-LFP coherences (Figure 2F) recorded under cannabinoid and control conditions across the population, we used a two-tailed paired t-test, pairing the values obtained at each site.

Estimates of the spatiotemporal response functions (STRFs) were measured by reverse correlation of recorded spike trains with the visual stimulus (the pseudorandom checkerboards described above). Each STRF estimate was generated by drawing 20 bootstrap samples (sampling with replacement) from the available trials. Figures 3A, B and Supplementary Figures 2 and 3 in File S1 show the mean of 20 such samples. For display, STRFs were normalized by mean-subtraction followed by division by the maximum of the absolute value of the response. The temporal response profile was defined as the spatial variance (mean square) of the STRF at each temporal lag, normalized to its peak. Response dynamics (peak latency and duration) were determined from the mean and standard deviation of best-fitting Gaussian. This process was repeated for each of the 20 bootstrap samples to yield the confidence intervals shown in Figures 3C and D.

To quantify extracellular spike waveforms, we used the time from peak to trough [31], [32]. The recordings in this study were part of a larger accumulated laboratory database of over 1000 extracellular waveforms. Consistent with the above authors, we found that the distribution of the peak-to-trough time in this database was bimodal (p<0.01 by the Hartigan dip test [33]), and used this bimodality to classify extracellular waveforms as fast (<405 microsec), slow (>430 microsec), or indeterminate.

## Supporting Information

File S1 Supplementary Figure 1, CP55940 alters dynamics of neuronal populations. (A) Average EEG power spectra before (black) and after (green) CP55940 administration, in two animals, L65 and L68. Heavier segments show frequencies which are significantly different (two group test within each animal, p<0.05). (B) Average LFP power spectra and (C) average LFP-LFP coherence in 2 animals, L69 (solid) and L72 (dashed) lines in the control condition (black) and following cannabinoid administration (green). Heavier segments show frequencies which are significantly different (paired t-test, p<0.05). Supplementary Figure 2, Further examples of units whose spatiotemporal response functions are altered by CP55940. Units: Units: L68t3.a, L68t3.d, L64t3.c, L65t1.b, L69t1.c, and L72t4.e. Supplementary Figure 3, Examples of units whose receptive fields are not altered by CP55940. Units: L64t3.b, L69t4.a and L69t3.c. Supplementary Figure 4, Firing rates during visual stimulation before (abscissa) and after (ordinate) CP55940 administration. Cells with shift in RF peak time of >3 ms shown in red.
(ZIP)
