# Supplementary figures and images for: Cannabinoid Neuromodulation in the Adult Early Visual Cortex
Source: PLoS One. 2014 Feb 19;9(2):e87362. doi: 10.1371/journal.pone.0087362 (PMC3929390; doi:10.1371/journal.pone.0087362)

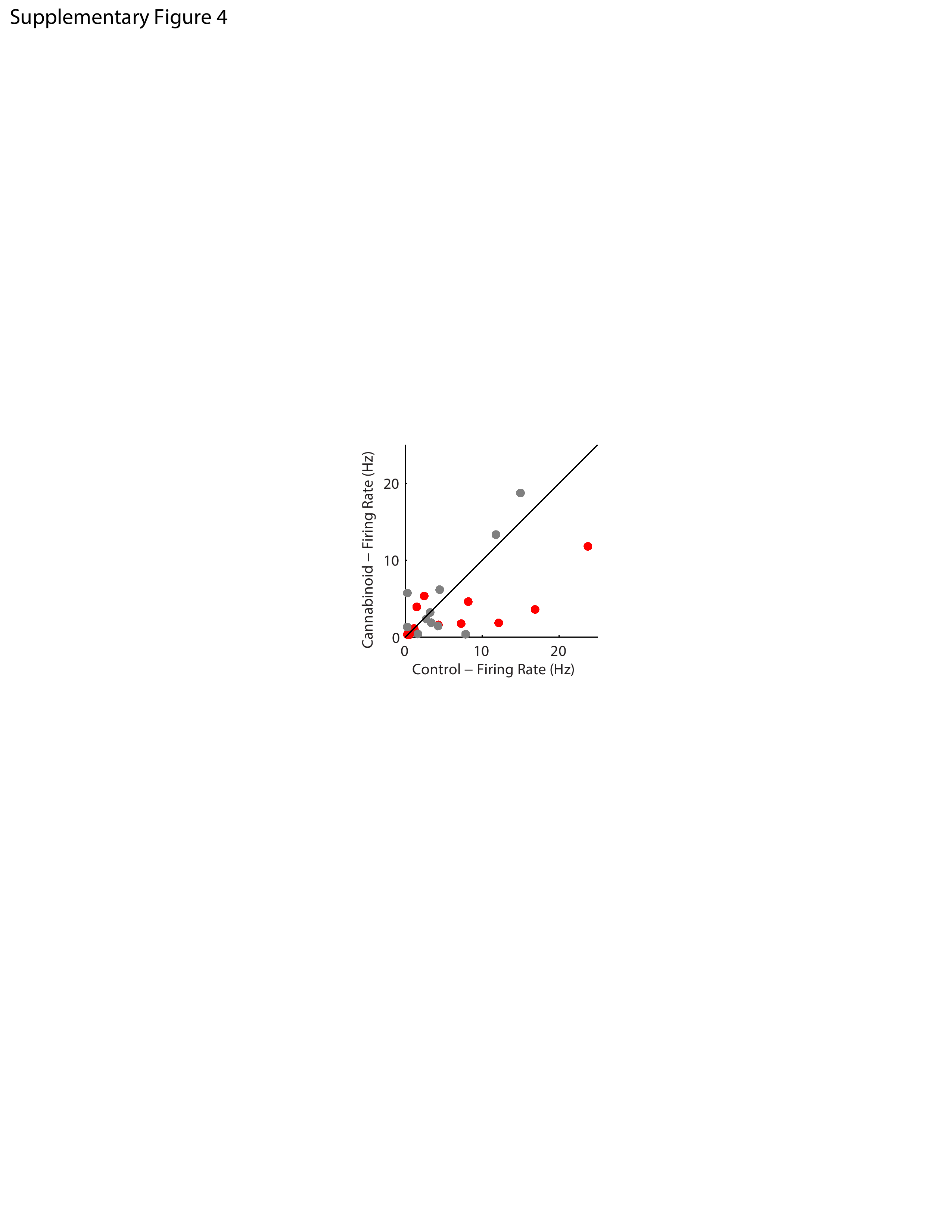

Supplement: File S1 — Supplementary Figure 1, CP55940 alters dynamics of neuronal populations. (A) Average EEG power spectra before (black) and after (green) CP55940 administration, in two animals, L65 and L68. Heavier segments show frequencies which are significantly different (two group test within each animal, p<0.05). (B) Average LFP power spectra and (C) average LFP-LFP coherence in 2 animals, L69 (solid) and L72 (dashed) lines in the control condition (black) and following cannabinoid administration (green). Heavier segments show frequencies which are significantly different (paired t-test, p<0.05). Supplementary Figure 2, Further examples of units whose spatiotemporal response functions are altered by CP55940. Units: Units: L68t3.a, L68t3.d, L64t3.c, L65t1.b, L69t1.c, and L72t4.e. Supplementary Figure 3, Examples of units whose receptive fields are not altered by CP55940. Units: L64t3.b, L69t4.a and L69t3.c. Supplementary Figure 4, Firing rates during visual stimulation before (abscissa) and after (ordinate) CP55940 administration. Cells with shift in RF peak time of >3 ms shown in red. (ZIP) [file pone.0087362.s001.zip › Supplementary Figure 4.tif]

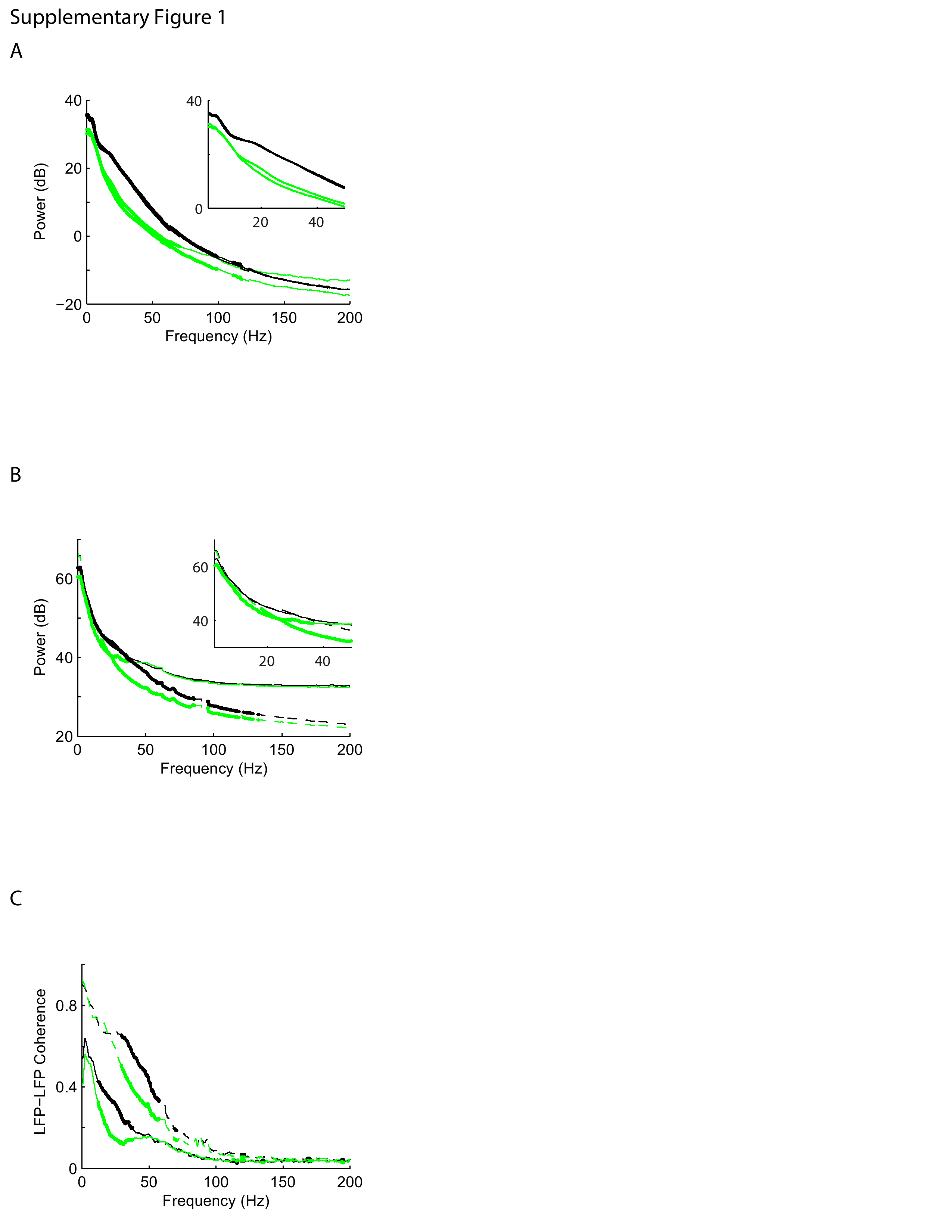

Supplement: File S1 — Supplementary Figure 1, CP55940 alters dynamics of neuronal populations. (A) Average EEG power spectra before (black) and after (green) CP55940 administration, in two animals, L65 and L68. Heavier segments show frequencies which are significantly different (two group test within each animal, p<0.05). (B) Average LFP power spectra and (C) average LFP-LFP coherence in 2 animals, L69 (solid) and L72 (dashed) lines in the control condition (black) and following cannabinoid administration (green). Heavier segments show frequencies which are significantly different (paired t-test, p<0.05). Supplementary Figure 2, Further examples of units whose spatiotemporal response functions are altered by CP55940. Units: Units: L68t3.a, L68t3.d, L64t3.c, L65t1.b, L69t1.c, and L72t4.e. Supplementary Figure 3, Examples of units whose receptive fields are not altered by CP55940. Units: L64t3.b, L69t4.a and L69t3.c. Supplementary Figure 4, Firing rates during visual stimulation before (abscissa) and after (ordinate) CP55940 administration. Cells with shift in RF peak time of >3 ms shown in red. (ZIP) [file pone.0087362.s001.zip › Supplementary Figure 1.tif]

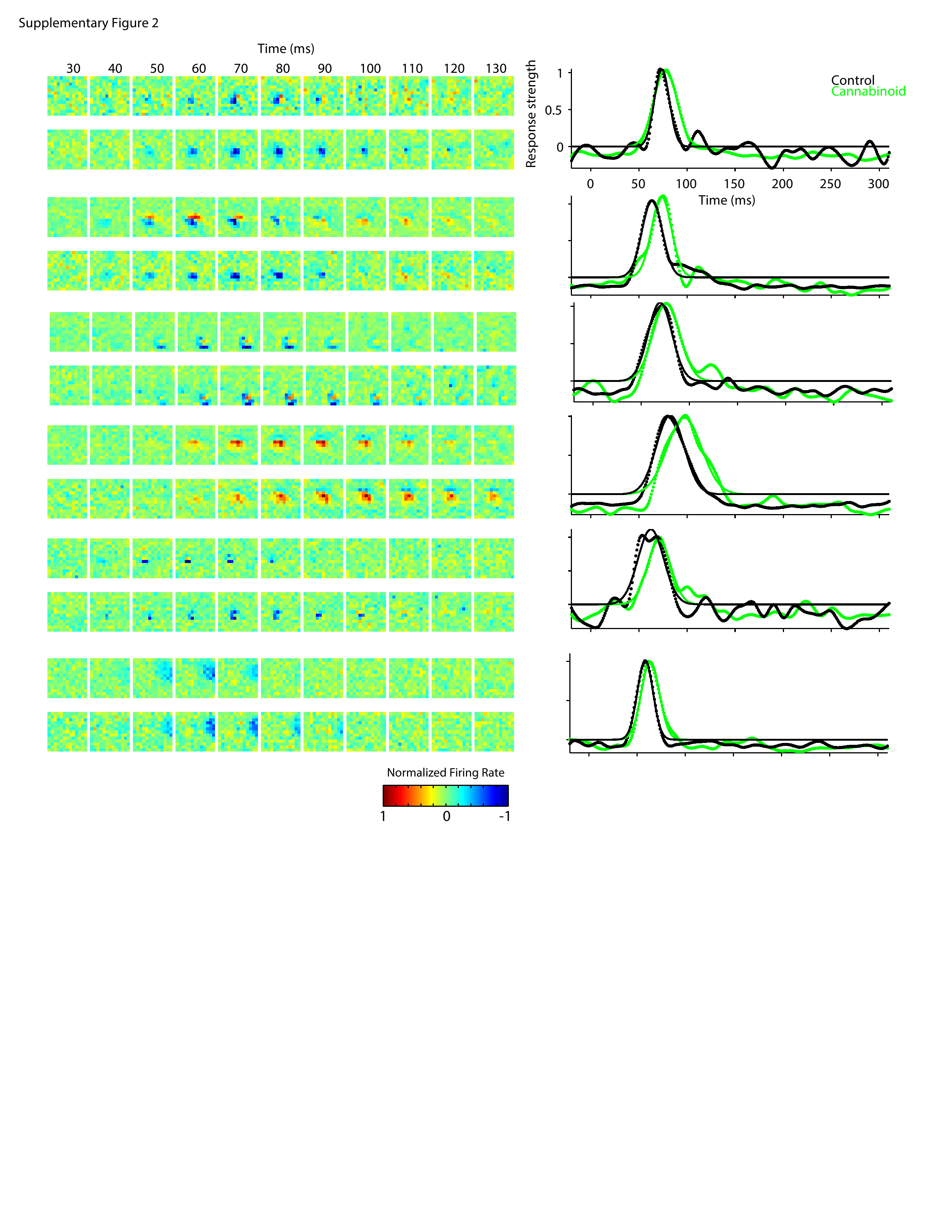

Supplement: File S1 — Supplementary Figure 1, CP55940 alters dynamics of neuronal populations. (A) Average EEG power spectra before (black) and after (green) CP55940 administration, in two animals, L65 and L68. Heavier segments show frequencies which are significantly different (two group test within each animal, p<0.05). (B) Average LFP power spectra and (C) average LFP-LFP coherence in 2 animals, L69 (solid) and L72 (dashed) lines in the control condition (black) and following cannabinoid administration (green). Heavier segments show frequencies which are significantly different (paired t-test, p<0.05). Supplementary Figure 2, Further examples of units whose spatiotemporal response functions are altered by CP55940. Units: Units: L68t3.a, L68t3.d, L64t3.c, L65t1.b, L69t1.c, and L72t4.e. Supplementary Figure 3, Examples of units whose receptive fields are not altered by CP55940. Units: L64t3.b, L69t4.a and L69t3.c. Supplementary Figure 4, Firing rates during visual stimulation before (abscissa) and after (ordinate) CP55940 administration. Cells with shift in RF peak time of >3 ms shown in red. (ZIP) [file pone.0087362.s001.zip › Supplementary Figure 2.tif]

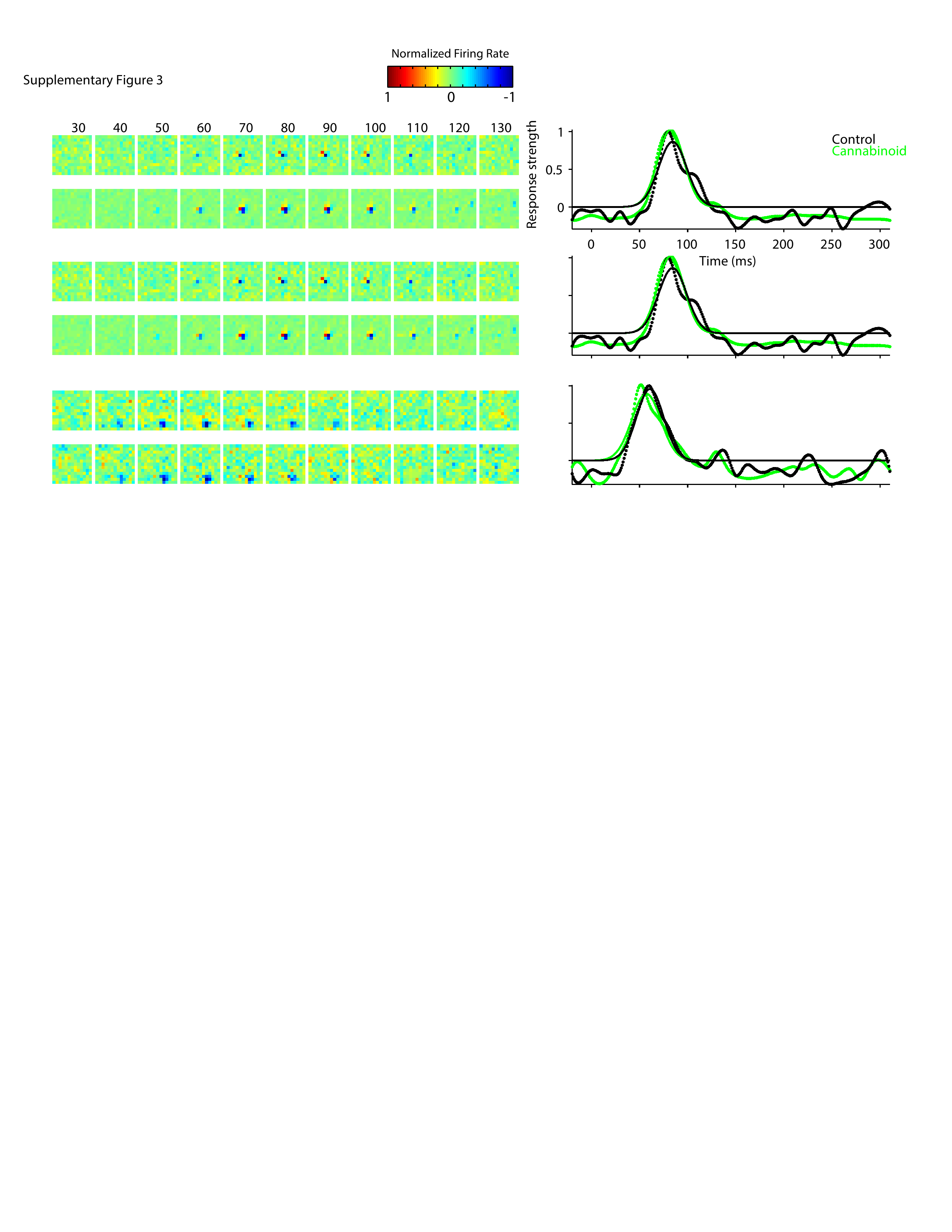

Supplement: File S1 — Supplementary Figure 1, CP55940 alters dynamics of neuronal populations. (A) Average EEG power spectra before (black) and after (green) CP55940 administration, in two animals, L65 and L68. Heavier segments show frequencies which are significantly different (two group test within each animal, p<0.05). (B) Average LFP power spectra and (C) average LFP-LFP coherence in 2 animals, L69 (solid) and L72 (dashed) lines in the control condition (black) and following cannabinoid administration (green). Heavier segments show frequencies which are significantly different (paired t-test, p<0.05). Supplementary Figure 2, Further examples of units whose spatiotemporal response functions are altered by CP55940. Units: Units: L68t3.a, L68t3.d, L64t3.c, L65t1.b, L69t1.c, and L72t4.e. Supplementary Figure 3, Examples of units whose receptive fields are not altered by CP55940. Units: L64t3.b, L69t4.a and L69t3.c. Supplementary Figure 4, Firing rates during visual stimulation before (abscissa) and after (ordinate) CP55940 administration. Cells with shift in RF peak time of >3 ms shown in red. (ZIP) [file pone.0087362.s001.zip › Supplementary Figure 3.tif]
